# Osteosarcoma following single fraction radiation prophylaxis for heterotopic ossification

**DOI:** 10.1186/1748-717X-7-140

**Published:** 2012-08-21

**Authors:** Michael K Farris, Varun K Chowdhry, Sheila Lemke, Mary Kilpatrick, Michael Lacombe

**Affiliations:** 1 Department of Radiation Oncology, SUNY Upstate Medical University, 750 East Adams Street, Syracuse, NY, 13210, USA; 2 Department of Medicine, SUNY Upstate Medical University, Syracuse, NY, 13210, USA

## Abstract

Radiotherapy for prophylaxis of heterotopic ossification (HO) is commonly used in high risk patients following orthopedic surgery. While treatment is effective and can prevent morbidity associated with HO, with any dose of radiation there is a concern of a radiation induced malignancy. Here we a report a case of radiation induced osteosarcoma which developed 11 years after a single fraction of 700 cGy. We performed dosimetric analysis by superimposing the patient’s original treatment field on a CT scan performed after the diagnosis. The radiotherapy dose for this patient is lower than classically reported for radiation induced sarcomas. We identified greatest bony destruction that was thought to be the epicenter of the tumor, and this was specially contoured on the diagnostic CT scan. This volume appears to be located at the edge of the radiotherapy field. Fifty percent of the treated volume received 240 cGy, the mean dose was 333 cGy. There was a variation across the treatment volume, between 21.8 cGy and 717 cGy. While a rare complication, we stress the importance of informing regarding the risk of a radiation induced malignancy following HO prophylaxis.

## Introduction

Heterotopic ossification (HO) of soft tissues following traumatic fracture, orthopedic surgery, or central nervous system injury, is a well known phenomenon described as early as 1883 [1]. Although not entirely understood, the pathogenesis is thought to involve stimulation of skeletal growth factors, possibly prostaglandins and bone morphogenic proteins, which then orchestrate the improper development of pluripotent mesenchymal cells towards ectopic production of mature bone in the tissues surrounding joints [2-5]. Usually HO presents as an incidental radiological finding, or painless joint stiffening. More severe cases may be painful, show signs of inflammation, or significantly impact mobility [2,6]. The reported incidence is highly variable. In high risk individuals following total hip arthroplasty for example, the incidence of HO has been reported as high as 90% [7].

The most common areas of HO development are around the femoral neck and greater trochanter following hip arthroplasty. Patients with a history of previous HO in either hip, bilateral hypertrophic osteoarthritis, or post-traumatic arthritis with hypertrophic osteophytosis are considered high risk patients. Men have a twofold higher incidence than women [8].

The idea that RT could be used to prevent HO is based largely on the assumption that osteoprogenitor cells present in soft tissues in the beginning phases of HO, would be highly mitotic and therefore sensitive to RT. In 1981 Coventrey et al. concluded that RT was an effective means of HO prevention [9,10].

The potential downsides to RT however, include effects on fertility, and the possibility of radiation induced malignancy. Although rare, radiation induced sarcoma (RIS) has been associated with RT for various diseases at an incidence of 0.09 and 0.11% [11]. The single 700 cGy fraction typically used for HO prevention however, was until recently, considered far lower than the doses commonly believed necessary to induce sarcomatous development [12-14]. To our knowledge, Mourad et al. is the only group to report a case of RIS following HO prophylaxis with two widely separated fractions to a total dose of 1400 cGy. Here we a second case of RIS which developed 11 years after only a single fraction of 700 cGy [14].

## Case report

### Initial presentation

A 26-year old Caucasian man sustained multiple injuries in a motorcycle accident in 2001. He presented with a right posterior hip dislocation and acetabular fracture as well as an open right distal tibia and fibular fracture which necessitated a below-knee amputation (BKA). He was treated with open reduction internal fixation (ORIF), and within 72 hours, received post operative prophylactic RT to the right hip including the acetabulum, femoral head and neck, as well as the greater trochanter. Treatment involved an open 8 × 15 field using anteroposterior-posteroanterior (AP/PA) 6-MV photons to 7 Gy in one fraction, without bone shielding.

The patient tolerated RT well. He did have multiple revisions to the base of his right residual limb over the years for episodes of poor wound healing and necrosis, but otherwise had an uneventful course until three years after RT, when he presented with a shooting painful neuroma of the right BKA stump and femoral nerve palsy. He did well following stump revision and neuroma excision with intramedullary replacement of nerve endings, and his femoral nerve palsy resolved.

### Presentation of radiation induced sarcoma

Seven years later, now 37-years old, the patient began to experience severe shooting pain along his right residual extremity. This had started just 10 hours after switching to a new prosthesis, but the pain persisted even on return to his old prosthesis, and radiographs showed several benign appearing proximal tibial and distal femoral bony cysts. He returned to the operating room for a BKA stump revision and tibal/peroneal neurectomy revision.

At the time of discharge, he began to notice a constant right sided hip pain which rapidly worsened over the next four months. The pain eventually became so intolerable that he had to remain bed ridden for three weeks, until he presented to the ED. A few days prior to presentation, he had noticed a palpable mass in the right hip. When positioned upright, he was most uncomfortable, and his pain localized primarily to the right hip and medial thigh. The joint maintained its usual range of motion, and he denied any fever, chills, nightsweats, warmth or erythema. He had lost 30 pounds over the preceding four months and described decreased appetite and energy levels. His only pertinent lab findings were mildly elevated CRP and ESR.

A plain film of the hip was read as unchanged from previous x-rays, however, magnetic resonance imaging (MRI) of the pelvis revealed a large soft tissue mass in the right iliac wing measuring 15 × 14 × 15.4 cm with intra-pelvic and extra-pelvic extension. Abdominal computed tomography (CT) also exposed additional sclerotic foci within the proximal right femur and sub-centimeter low density lesions within the posterior right hepatic lobe. Right external iliac and retroperitoneal lymph nodes were not pathologically enlarged. A chest CT showed no lung metastases, and a bone scan displayed only activity in the right pelvic gluteal region.

Core biopsy of mass revealed high grade sarcoma with cartilaginous differentiation suggestive of chondroblastic osteosarcoma. The sample consisted of pleomorphic spindle cells in a fibrous background containing eosinophilic glassy osteoid like material. Immunohistochemisty studies were positive for S-100, CD34, and negative for CD99. Both Neuronspecific enolase (NSE) and CD57 showed patchy positivity within the cartilaginous and spindle cell areas.

His disease was staged as T2 N0 M0 G3, stage IIB, and the treatment plan included six cycles of cisplatin and adriamycin. After completing the first two cycles his pain noticeably improved. He continues to tolerate chemotherapy reasonably well and the operational status of his mass will be reassessed radiologically for possibly curative surgery in the near future.

## Discussion

Although HO is a benign disease, its propensity to adversely affect quality of life, makes prophylactic treatment appealing. Radiation therapy (RT) was first considered for HO prophylaxis as early as the 1950’s when Cooley et al. experimented with its effects on bone repair [15]. In 1971, Craven et al. proposed that osteoprogenitor cells present in the initial stages of HO development, were exquisitely sensitive to RT due to a high mitotic rate [9]. Coventrey et al. concluded RT was an effective means of HO prevention. With a retrospective analysis of post operative total hip arthroplasty patients, they showed that only 19% of patients developed significant HO following RT [10]. To maintain efficacy, treatment is generally given within 4 days of surgical intervention based on studies by Sylvester et al. After this window has elapsed efficacy of RT drops precipitously [16]. The effectiveness of a single fraction to 7 Gy has been determined equivalent to higher doses or multiple fractions [17]. Healy et al. illustrated that HO developed in only 10% of patients receiving 7 Gy whereas 63% of patients receiving 5.5 Gy developed HO [18]. A single fraction of 7 Gy has therefore become common practice.

Cahan et al. developed the criteria for defining RIS which initially included; neoplastic growth within the RT field, no pre-existing bone malignancy, histologic confirmation, and a latency period of at least 5 years. Arlen et al. then modified these criteria to include; neoplasms in the peri-irradiated areas, bone without a primary malignant osteoblastic lesion when the RT was given, and also included tumors diagnosed earlier than 5 years from RT. Some authors have suggested the latency period for RIS should be considered as short as 1–6 months following RT [13,19,20].

The first and only other reported case of RIS associated with HO prophylaxis, was discovered in a study by Mourad et al. spanning 18 years involving 1,724 traumatic fracture patients treated with RT to 7 Gy post ORIF, providing an incidence rate of 0.058. That patient had been irradiated 15 years earlier with a single 7 Gy fraction at 37 years old, but he ultimately developed debilitating HO in the treated hip, and was subsequently treated with surgery as well as a second 7 Gy RT fraction. Sixteen months later, he presented similarly to our patient, with progressive thigh pain and soft tissue swelling. Although treated aggressively, that patient died as a result of lung metastasis shortly following diagnosis [14,21].

Brady et al. proposed that the presence of metastasis, the extent of surgical resection, and the primary tumor size before resection, could all be viewed as unfavorable prognostic factors for RIS [22]. In general RIS originating at any site reportedly tends toward a worse outcome than sporadic sarcomas [23]. The cumulative 5 year disease free survival is often reported in the range of 10-30% and the median survival approaches one year [24]. A recent article by Bjerkenhagen et al. suggested a poorer prognosis of RIS is likely due to a higher incidence of central tumor site, difficult or incomplete surgical resection based on location, microscopic tumor necrosis, and a higher incidence of distant metastases [25]. Moreover, some authors have suggested that fibrotic tissue changes resulting from previous RT would prevent chemotherapy from reaching target areas in RIS effectively [26]. The relative rarity of RIS, likely makes definitive conclusions on comparative prognosis difficult. Often RIS presents insidiously. In the case of HO prophylaxis, typical followup has involved only periodic radiographs [14]. To the best of our knowledge, there is no standard followup protocol in place, but as illustrated in the above case, simple radiographs may lack adequate sensitivity for detection of large masses. While early CT or MRI, would undoubtedly improve sensitivity, they may not be feasible for all patients, given the low incidence of RIS.

Pakos et al. suggested that the low incidence of RIS following HO prophylaxis may be partly due to a primarily older patient population receiving treatment. These patients simply do not survive long enough to develop RIS. In a study involving 1143 patients, the median age at treatment was 61 [27]. Perhaps as younger patients are followed longer, we may begin to see an increased incidence of RIS.

RIS has been associated at an estimated incidence of 0.09 - 0.11% considering all cases of RT [11]. Mark et al. reported an absolute risk of 0.03 - 0.8% where RT was used to treat gynecologic malignancies [28]. Osteosarcomas tend to be the most common type of RIS, followed closely by fibrosarcoma [11,22,29].

It was previously believed that the development of RIS likely required a minimum dose of at least 30–40 Gy, and that doses of 55 Gy or more are associated with increased risk of RIS [11,14,19]. A noteworthy point in this report is that the dose for RIS in this patient is far lower than what is classically reported in the literature. Based on data from atomic bomb survivors, there is a linear dose response relationship between radiation dose and the incidence of carcinomas from 0.1 Sv to 2.5 Sv. While low-dose exposure increases carcinoma risk, sarcomas are typically observed in regions of tissue that received higher doses of radiotherapy, either within or at the edge of the radiotherapy field [30,31]. The patient presented in this report received only one 7 Gy fraction to to the surgical area, and his RIS subsequently developed on the edge of the treatment field 11 years later.

**Figure 1:**
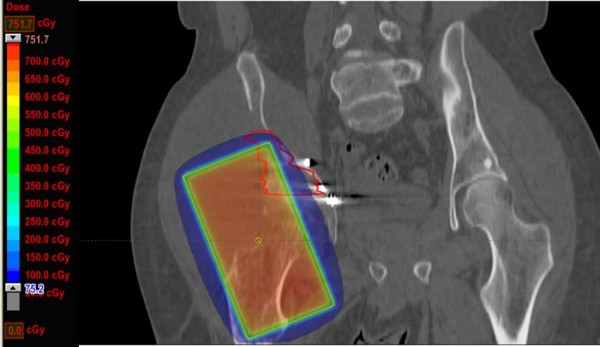
**The original 8 × 15 field was digitally reconstructed and superimposed on a CT scan following the patient’s diagnosis of osteosarcoma, and the treatment plan was subsequently recreated.** The region of greatest bony destruction was thought to be the epicenter of the tumor, and was specially contoured on the diagnostic CT scan. The volume appears to be located at the edge of the radiotherapy field, including both areas that received >700 cGy and <100 cGy.

**Figure 2:**
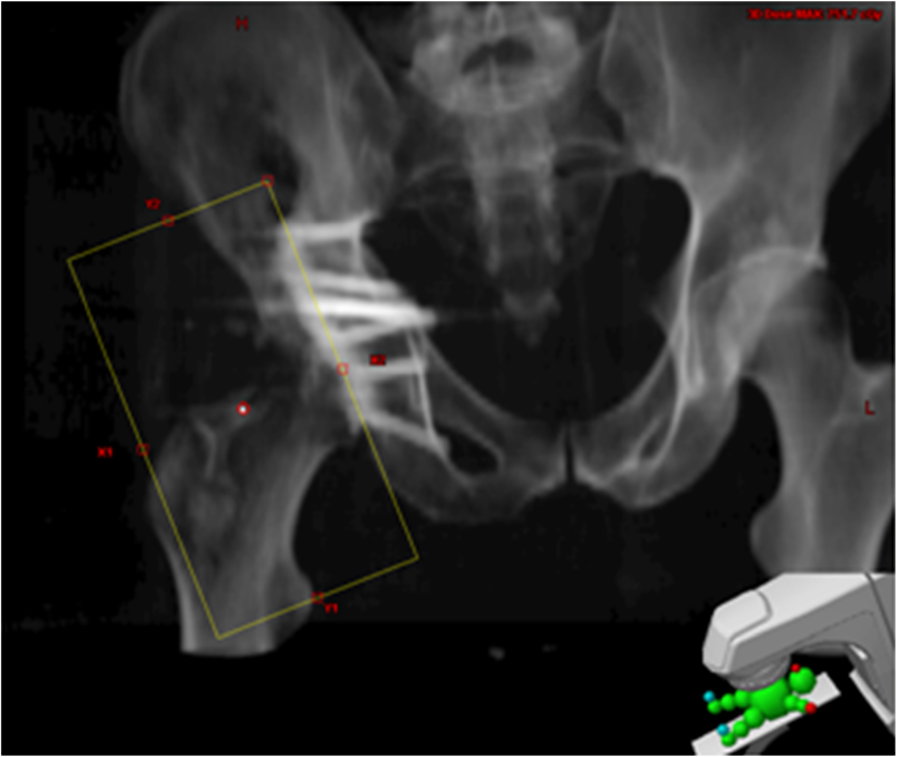
**Digitally reconstructed radiograph (DRR) based on original portal images.** Portal images from patient’s original treatment were recreated and superimposed on CT scan obtained following the diagnosis of osteosarcoma. An open 8 × 15 field was utilized for patient. Treatment was delivered using 6 MV photons, with a source to skin distance (SSD) or 91.5 cm.

**Figure 3:**
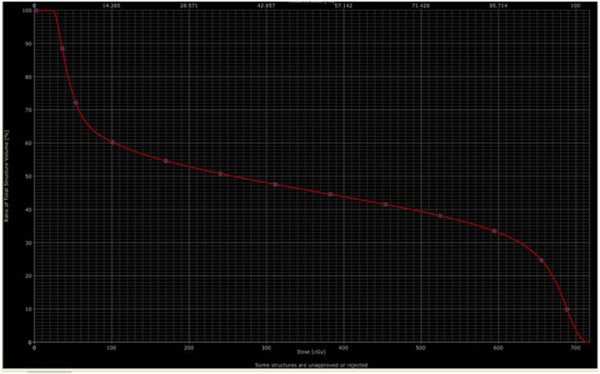
**Dose-volume histogram of the contoured volume, including the areas of greatest bony destruction.** Fifty percent of the treated volume received 240 cGy, although there was a wide variation across the treatment volume, from a maximum of 717 cGy to a minimum of 21.8 cGy with a mean of 333 cGy.

The original 8 × 15 field was superimposed on a current CT scan of the patient’s sarcoma Figure 1, and the digitally reconstructed radiograph is shown in Figure 2. His treatment plan was subsequently recreated using Varian treatment planning software. The region of greatest bony destruction was thought to be the epicenter of the tumor, and was specially contoured on the diagnostic CT scan. This volume appears to be located at the edge of the radiotherapy field, including areas that both received both >700 cGy and < 100 cGy. Fifty percent of the treated volume received 240 cGy, although there was a wide variation across the treatment volume, from a maximum of 717 cGy to a minimum of 21.8 cGy with a mean of 333 cGy. The dose-volume-histogram (DVH) which was developed based on the contoured volume is shown in Figure 3.

Although RIS remains a rare treatment complication, there are several other disadvantages of RT in this setting including considerable costs, and scheduling difficulties given the narrow 3–4 day treatment window. Post operative pain and immobilization can also prove to be significant boundaries to the precise positioning necessary for treatment. Radiation’s impact on fertility has also been a concern in younger patients, however Patel et al. showed that the majority of testicular dose can be shielded against effectively [32].

Non-steroidal anti-inflammatory drugs (NSAIDs) can serve as an alternative method of HO prophylaxis presumably through systemic inhibition of prostaglandins that promote osteoprogenitor development. A typical and effective regimen is Indomethacin 25–50 mg three times a day, for 6 weeks, although other NSAIDs and different dosage schedules may be used. Compared to RT, NSAIDs are a considerably less expensive option, but patients often have poor compliance due to gastritis or other GI complications. There is also a more significant incidence of nonunion with NSAIDs, a problem that can be avoided by shielding prostheses in RT [2]. Post surgical use of NSAIDs also produces considerable bleeding risks given these patients are usually anti-coagulated with heparin or warfarin for deep venous thrombosis prophylaxis [27]. Pakos et al. also compared effectiveness of NSAIDs vs RT in a large meta analysis and found RT to be slightly superior at preventing the most severe cases of HO development, however the absolute difference was very small at only 1.2% [27].

## Conclusions

Radiotherapy remains an effective method of prophylaxis for HO in high risk patients. We have presented the above case to increase awareness in literature of this rare complication following HO prophylaxis. Younger patients have an increased risk of developing a secondary malignancy with radiotherapy, and it is possible that we may see an increased frequency of RIS in the future. The difficulty of early detection and a generally poor prognosis of RIS suggest that imaging follow-up protocols may be reasonable in younger patients. Furthermore, it is important to inform patients regarding the risk of a radiation induced malignancy. As many of these patients are on narcotic pain medications post-operatively, when possible, radiation oncology consultation should be obtained prior to surgery so that patients can be best informed of the risks and benefits of treatment. Based on the reported case, we suggest that prior to recommending prophylactic RT for young patients at risk for HO, the potential risks should be weighed strongly against the benefits.

## Consent

We obtained written informed consent from the patient publication of this report and any accompanying images.

## Abbreviations

HO: Heterotopic ossification; RT: Radiation therapy; RIS: Radio induced sarcoma; ORIF: Open reduction internal fixation; BKA: Below knee amputation; MRI: Magnetic resonance imaging; CT: Computed tomography; NSE: Neuron specific enolase; NSAID: Non steroidal anti-inflammatory drugs; DRR: Digitally reconstructed radiograph; SSD: Source to skin distance; AP/PA: Anterior posterior/Posterior anterior; Sv: Sievert; cGy: Centigray.

## Competing interest

The authors have no competing interest to disclose.

## Authors’ contribution

MKF, VLC, ML drafted initial manuscript. SL wrote and contributed to the section regarding the patient’s re-presentation and treatment for the sarcoma. MK re-created treatment plan from initial radiotherapy course and contributed to the dosimetric analysis section. All authors read and approved the final manuscript.
